# Signaling and Transcription Factors during Inner Ear Development: The Generation of Hair Cells and Otic Neurons

**DOI:** 10.3389/fcell.2017.00021

**Published:** 2017-03-24

**Authors:** Héctor Gálvez, Gina Abelló, Fernando Giraldez

**Affiliations:** Developmental Biology, CEXS, Parc de Recerca Biomèdica de Barcelona, Universitat Pompeu FabraBarcelona, Spain

**Keywords:** atoh1, Neurog1, Hes and Hey factors, Notch signaling pathway, cell fate specification, hair cell regeneration

## Abstract

Integration between cell signals and bHLH transcription factors plays a prominent role during the development of hair cells of the inner ear. Hair cells are the sensory receptors of the inner ear, responsible for the mechano-transduction of sound waves into electrical signals. They derive from multipotent progenitors that reside in the otic placode. Progenitor commitment is the result of cell signaling from the surrounding tissues that result in the restricted expression of SoxB1 transcription factors, Sox2 and Sox3. In turn, they induce the expression of Neurog1 and Atoh1, two bHLH factors that specify neuronal and hair cell fates, respectively. Neuronal and hair cell development, however, do not occur simultaneously. Hair cell development is prevented during neurogenesis and prosensory stages, resulting in the delay of hair cell development with respect to neuron production. Negative interactions between Neurog1 and Atoh1, and of Atoh1 with other bHLH factors driven by Notch signaling, like Hey1 and Hes5, account for this delay. In summary, the regulation of Atoh1 and hair cell development relies on interactions between cell signaling and bHLH transcription factors that dictate cell fate and timing decisions during development. Interestingly, these mechanisms operate as well during hair cell regeneration after damage and during stem cell directed differentiation, making developmental studies instrumental for improving therapies for hearing impairment.

## The induction of neural competence in the otic placode

The ear is one major sensory organ of the vertebrate head that is responsible for the senses of hearing, balance and acceleration. The vertebrate inner ear derives from the otic placode, a thickening of the head ectoderm. The formation of the inner ear requires a series of cell fate decisions and morphogenetic events with a precise temporal and spatial pattern (Fritzsch et al., 2006; Groves and Fekete, 2012). Mature sensory organs of the vestibular and auditory regions of the inner ear are formed by three cells types: hair cells (HC), supporting cells (SC), and neurons, which in amniotes derive from a common neurosensory pool of cells.

One crucial step during inner ear development is the specification of neurosensory progenitors and the diversification of the different cell types. This is probably the first developmental decision in the otic epithelium and it reflects the segregation of two functionally independent domains, one with neurosensory competence and another devoid of it (Abello and Alsina, 2007). The neurosensory domain gives rise to otic sensory neurons, sensory hair cells and supporting cells in chick and mouse (Satoh and Fekete, 2005; Raft et al., 2007). The expression of Sox3 and Sox2, Fgf10, and also that of members of the Notch pathway like Delta1, Hes5, and Lunatic Fringe is restricted to the neurosensory domain (Abelló et al., 2007). SoxB1 genes have a proneural function (See Box 1) and drive the expression of Neurog1 and Atoh1 (Jeon et al., 2011; Neves et al., 2012). The complementary non-neural domain shows two major patterning genes, Lmx1b and Iroquois1, and two members of the Notch pathway, Serrate1 and Hes1 (Abelló et al., 2007, 2010).

Box 1 **bHLH in vertebrates: has Atoh1 lost its proneural function?**

What is a proneural gene? A proneural gene must fulfill three main characteristics (Hassan and Bellen, 2000): First, its expression precedes and coincides with the selection of neuronal precursor cells. Secondly, its function is both necessary and sufficient for the specification of a given neuronal lineage in a cell autonomous fashion. Finally, its loss of function results in the deletion (and its missexpression ectopic development) of a given lineage. Proneural genes were first identified in Drosophila peripheral nervous system development. The Achaete–Scute complex (AS-C) genes were identified as proneural genes encoding bHLH factors. Later on, atonal (Atoh1 in mammals) was identified by PCR (Jarman et al., 1993). Atonal in Drosophila is the master gene for the formation of chordotonal organs, which are mechano-receptors of insect muscles. Atonal gene selects the progenitors that give rise to the mature organs. Atonal loss of function abolishes chordotonal organs and its missexpression favors their ectopic formation (Jarman et al., 1993). Are Atoh1 and Neurog1, the vertebrate homologs of atonal, also proneural genes? Atoh1 and Neurog1 overexpression drives, respectively, ectopic hair cell and neuron formation (Izumikawa et al., 2005; Evsen et al., 2013), and their loss of function results in the lack of hair cells or neurons (Ma et al., 1998; Bermingham, 1999). However, their function is far more restricted and, like in other regions of the Nervous System, they do not provide a broad neural competence, but a far more restricted lineage selection (for example, HCs and SCs in the case of Atoh1 and the inner ear). The broad neural competence is rather dependent on SoxB1 (Azuara et al., 2006; Puligilla and Kelley, 2017). This shows a proneural identity crisis in vertebrate development and the taking over by SoxB1 proteins (Hassan and Bellen, 2000).

FGF and BMP signaling differentially regulate the expression of Sox3 and Lmx1, and their respective restriction to the anterior and posterior domains (Abelló et al., 2007; Schneider-Maunoury and Pujades, 2007). The regionalization of the otic placode into neurosensory and non-sensory territories requires also the functional integrity of the Notch pathway for its stabilization (Abelló et al., 2007). The non-sensory region of the otic placode receives signals that confer posterior identity (Bok et al., 2011). Retinoic acid (RA), which is known to posteriorize the embryonic body axis, is also required to specify the posterior character of the otic placode. Expression of RA synthesizing and degrading enzymes coincides with the AP boundary of the otic placode, and experiments in chicken and zebrafish have disclosed a developmental window during which the otocyst receives and is sensitive to RA posteriorizing signals (Bok et al., 2011; Radosevic et al., 2011).

**Figure 1:**
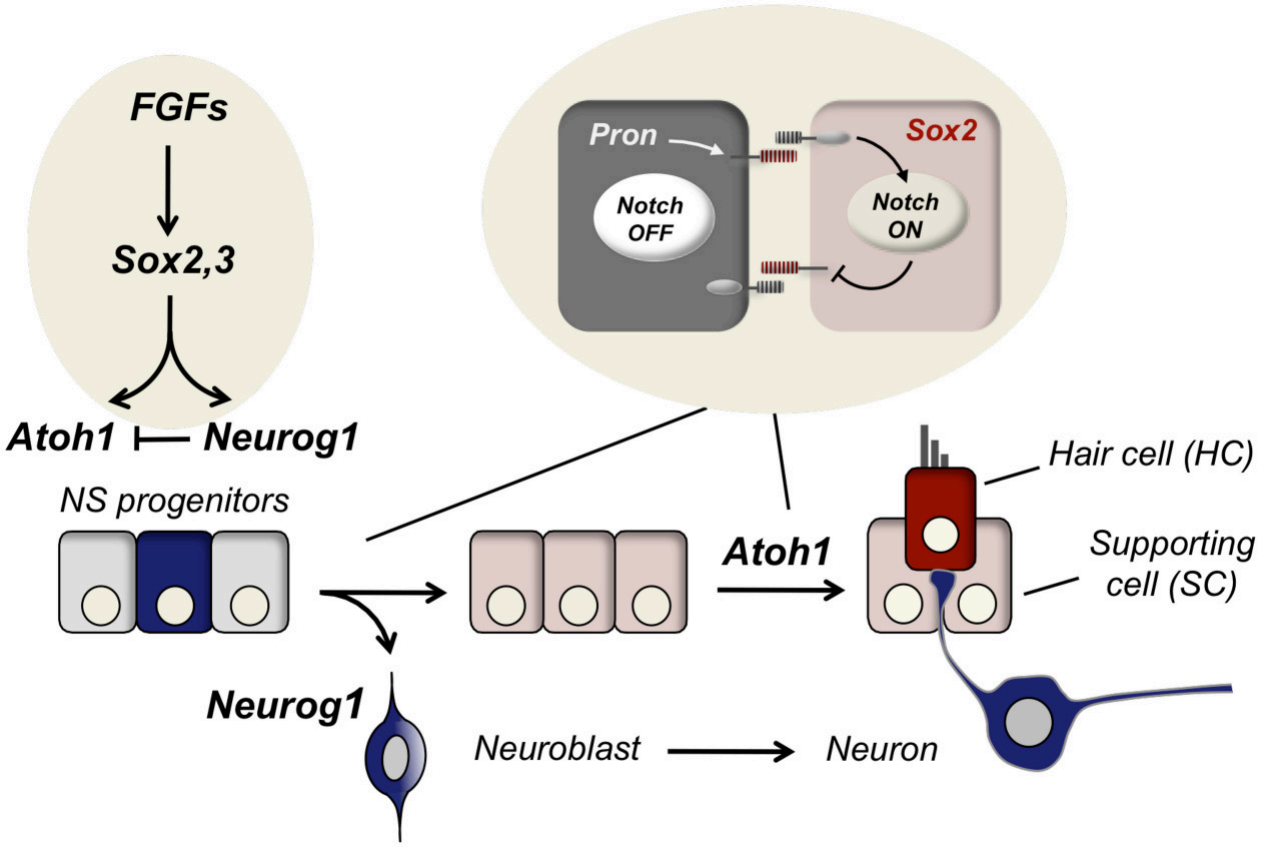
**Specification of neurons and hair cells during inner ear development**. The three cell types that constitute the functional unit of the sensory patches of the inner ear are: neurons (blue), supporting cells (SC, pink) and hair cells (HC, red). The three cell types derive from the neurosensory epithelium that is specified by Sox2 and Sox3 after FGF signaling. Upon neurosensory induction, HC differentiation is delayed with respect to neurons. Neurog1 is expressed in neuronal precursors and after neurogenesis Atoh1 is expressed in the prospective hair cells. Notch signaling contributes to cell determination in two rounds of lateral inhibition, first to single out neuronal precursors (neurogenesis) and, secondly, to decide between hair cells and supporting cells (sensorigenesis).

Two main cell fate decisions are made sequentially during ear development. First, neurosensory progenitors produce either neuronal (neuroblast) or sensory precursors. Secondly, once neurons have delaminated, the progenitors that remain in the epithelia develop into either hair cells or supporting cells. The differentiation of neurons and hair cells is driven by the expression of, respectively, Neurog1 and Atoh1, two basic Helix-Loop-Helix (bHLH) proteins. Notch signaling plays a critical role in these two sequential decisions because it is instrumental in forcing precursors to adopt alternative fates by the mechanism of lateral inhibition (Figure 1).

Evidence in different species suggests that neurosensory progenitors are multipotent. Lineage analyses by viral tracing in chicken embryos demonstrated that bipotential neurosensory progenitor cells are present in the otic placode (Satoh and Fekete, 2005) and dye-labeling of otic placode progenitors showed that neurons and hair cells derive from the neurosensory domain of the otic vesicle (Bell et al., 2008). Furthermore, genetic fate mapping in mouse and chick indicates that vestibular sensory hair cells derive from Sox2 expressing progenitors residing in the neurosensory domain of the otic placode (Raft et al., 2007; Neves et al., 2012). In zebrafish, there are three progenitor pools, one specific to neurons, another specific to hair cells and a third one that can give either neurons or hair cells until later stages (Sapède et al., 2012), but all come from a population that expresses Atoh1b, suggesting that they also may share a common progenitor (Millimaki et al., 2007).

## Sox2 and neural competence

Sox genes are transcription factors that belong to the High Moblity Group (HMG) box domain proteins (Kamachi and Kondoh, 2013). One subfamily of Sox genes is the SoxB group, which is split in turn into two sub-groups: SoxB1 that includes Sox1, Sox2, and Sox3, being only Sox2 and Sox3 expressed in the vertebrate inner ear (Neves et al., 2007; Abelló et al., 2010), and SoxB2 that comprises Sox14 and Sox21, which are transcriptional repressors (Kamachi and Kondoh, 2013) and from which only Sox21 is expressed during ear development (Freeman and Daudet, 2012).

Sox2 is critical for the specification of neurons and hair cells in the neurosensory domain of the otic placode (Kiernan et al., 2005; Neves et al., 2012). Sox2 is able to activate both Neurog1 and Atoh1, but it is downregulated in differentiated neurons and hair cells. Sox2 expression remains high in supporting cells, suggesting that this cell type retains progenitor properties (Neves et al., 2007; Evsen et al., 2013; Kamachi and Kondoh, 2013). The expression of Sox2 in the inner ear is driven by signals from the surrounding tissues. Like in other regions of the nervous system, FGF signaling is determinant for setting the onset of SoxB1 factors (Alsina et al., 2004; Sweet et al., 2011; Ono et al., 2014). In the inner ear, first Sox3 and then Sox2 expression depends on FGF signaling emanating from the underlying mesoderm, the hindbrain and probably from the otic placode (Schneider-Maunoury and Pujades, 2007; Groves and Fekete, 2012). Sox3, which is expressed in the neurosensory domain in chick is not detected in the mouse, where Sox9 is co-expressed along with Sox2 in the prosensory region (Mak et al., 2009).

## The regulation of Neurog1 and Atoh1

### The regulation of Neurog1

Neurog1 (Neurogenin1) is a basic helix-loop-helix (bHLH) transcription factor that behaves as master regulator for neuronal differentiation in different vertebrates (Henrique et al., 1997; Ma et al., 1998; Alsina et al., 2004; Evsen et al., 2013). Neurog1 is an Atonal-related protein (ARP; Hassan and Bellen, 2000). On average, it shares with Atoh1 53% amino acid identity in the bHLH domain, and differs from Atoh1 in four basic domain residues (Sommer et al., 1996). Three neurogenins have been described in mammals. Neurog1 and Neurog2 function as neuroblast selector genes in mouse (Ma et al., 1998), but in the chicken and mouse inner ear, only Neurog1 is expressed during ear development (Ma et al., 1996; Evsen et al., 2013).

Sox2 is necessary for Neurog1 up-regulation in the otic epithelium (Jeon et al., 2011; Neves et al., 2012). In mice, Neurog1 is also activated by Six1 and Eya1 that synergize with Sox2 (Zheng et al., 2003; Ahmed et al., 2012b). The neurosensory domain has high Notch activity, and Jeon and colleagues showed that the enhancer of Neurog1 is activated by high levels of NICD (Notch Intracellular Domain), while Atoh1 enhancer is not (Jeon et al., 2011). This may favor that Neurog1 expression precedes Atoh1 in the otic vesicle (Neves et al., 2011). However, later in development Notch signaling represses Neurog1 expression in the cells that remain in the epithelium.

Neurog1 expression is controlled by different cis-elements located 5′ and 3′ to the Neurog1 coding sequence. These enhancers drive the expression of Neurog1 in midbrain, hindbrain, trigeminal ganglia, and ventral neural tube. For Neurog1 expression in the dorsal neural tube only a 5′ enhancer has been identified (Nakada et al., 2004). Another enhancer region drives Neurog1 activity to the VIII cochlea-vestibular ganglion (Murray et al., 2000). The configuration of these enhancers is similar to the cis-elements identified for Neurog2 (Simmons et al., 2001), suggesting that there is a tight regulation of the two Neurogenins. Nakada et al. (2004) speculated that possibly the conservation between Neurog1 and Neurog2 arises from gene duplication. The modular organization of Neurogenins cis-regulatory regions contrasts with the single enhancer regulation described for Atoh1 (Helms et al., 2000 and see below).

### The regulation of Atoh1

**Figure 2:**
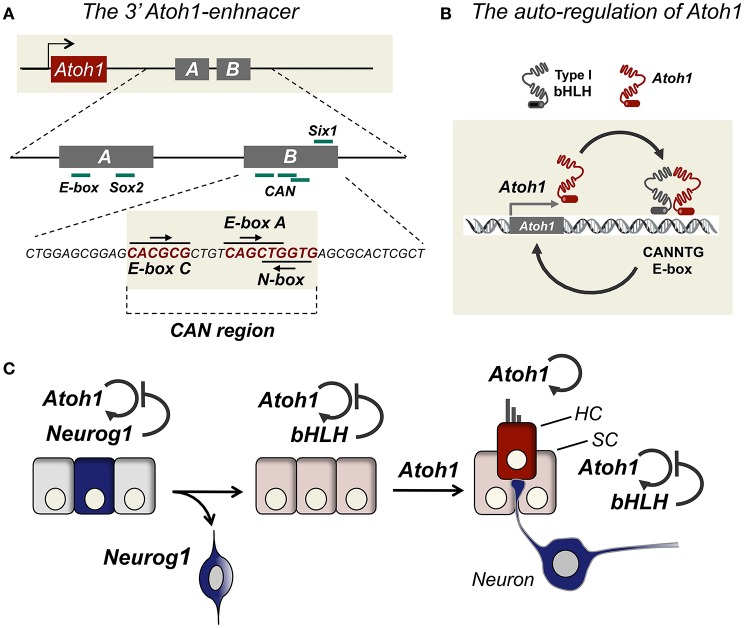
**The regulation of Atoh1 by signal integration in the 3′Atoh1 enhancer. (A)** The 3′Atoh1 enhancer is located 3.5 Kb downstream Atoh1 coding region and it consists of two enhancer named, Enhancer A and Enhancer B. Different transcription factors bind to this region like Sox2 in Enhancer A, and Six1 in Enhancer B. The three E-boxes in Enhancer B are putative sites for Atoh1 repression. **(B)** Atoh1 binds to the class A E-box located in the Enhancer B and is able to activate its own transcription. **(C)** During neurogenesis Atoh1 expression is silenced by Neurog1 and its expression is further delayed by the counteraction.

Atoh1 expression is regulated by a downstream enhancer, which depends on its interaction with Atoh1. In other words, Atoh1 expression relies on its auto-regulation. This implies that crucial events in the developmental regulation of Atoh1 are the chromatin arrangements that allow the interaction of Atoh1 with its own enhancer, and also the activity of potential repressors that break this loop (See BOX 2). Work by Jane Johnson's lab discovered a region in the Atoh1 genome landscape that recapitulates Atoh1 expression during mouse and chicken development (Helms et al., 2000). Transgene expression in mouse identified a region that directed the expression to the neural tube, external granular layer (EGL) of the cerebellum from rhombic lip, and the developing hair cells of the cochlea and semicircular canals. The region contains a 1.7 Kb fragment located 3.4 Kb 3′ of the Atoh1 coding region that recapitulates the expression of Atoh1. This region is called the 3′Atoh1-enhancer (see Figure 2A).

Box 2 **The priming of Atoh1.**

Priming of Atoh1 in the developing ear may be also promoted by other mechanisms rather than Atoh1 autoactivation. Changes in histone modifications can change the transcriptional hierarchy that controls cell differentiation (Azuara et al., 2006). The dynamic changes in the histone modifications H3K4me3/H3K27me3, H3K9ac, and H3K9me3 indicate that there is a progression from poised to active, and finally to repressive marks in the Atoh1 locus. This sequence correlates with the onset of Atoh1 expression and its subsequent silencing during the perinatal period (Stojanova et al., 2015). The inhibition of acetylation blocks Atoh1 mRNA expression in nascent hair cells, as well as the ongoing hair cell differentiation during embryonic organ of Corti development (Stojanova et al., 2015). Contrarily, histone deacetylase inhibition favors the expression of hair cell markers in mouse utricle progenitor cells (Hu and Wang, 2014). Cochlear explants treated with histone deacetylase inhibitor increase the levels of Atoh1 mRNA in early post-natal mice (Stojanova et al., 2015). This suggests that Atoh1 is poised during developmental stages and thereby ready to be activated. However, after birth the Atoh1 locus becomes methylated and cannot be transcribed when hair cells are damaged. The epigenetic status of Atoh1 locus during organ of Corti development shows a bivalent mark of the Atoh1 locus by H3K27me3 and H3K4me3, prior to the upregulation of Atoh1 (Stojanova et al., 2015). This is consistent with the idea that Sox2 poises/primes the Atoh1 locus until Atoh1 itself is able to bind to the E-box A and trigger Atoh1 expression.

Two regions within the 3′Atoh1-enhancer (3′Atoh1-enh) show a high homology between humans and mouse, and they were named Enhancer A and Enhancer B (EnhA and EnhB). The length of A and B is highly conserved in species like chicken, mouse, and human (Ebert, 2003), although the distance in between them varies among species. Interestingly, Helms et al. (2000) observed that transgenic embryos for Atoh1-enh/lacZ transgenic mice had no detectable β-gal activity in the Math1 null background, and this was shown to be also the case for the 3′Atoh1-enhancer-GFP reporter in the inner ear (Raft et al., 2007). This suggested that the activity of the 3′Atoh1-enh is dependent on Atoh1 expression and that autoregulation is one major mechanism for setting Atoh1 transcriptional activity (Figure 2B).

The 3′Atoh1-enh contains several E-boxes, which are six-nucelotide DNA sequences that bind bHLH proteins, like Atoh1, Neurog1, and Hes/Hey factors (CANNTG; (Massari and Murre, 2000)). The Enhancer A contains a degenerated E-box and the Enhancer B three E-boxes. E-boxes in Enhancer B are a class A, a class C and a reversed N-box. All three boxes are very close, and class A and N-box overlap (Figure 2A, the CAN region for C-, A- and N-boxes). As mentioned above, Helms et al. (2000) already identified the class A E-box located in Enhancer B as crucial for Atoh1 autoactivation. Besides the CAN region, the 3′Atoh1-enh region contains putative binding sites for a menagerie of transcriptional activators and repressors. Some of them like Sox2, Six1/Eya1, and β-catenin bind directly to the enhancer as shown by biochemical assay (Akazawa et al., 1995; Ebert, 2003; Mutoh et al., 2006; Briggs et al., 2008; D'Angelo et al., 2010; Shi et al., 2010).

Sox2 is sufficient to activate Atoh1 and to induce ectopic hair cell formation in the chick otocyst (Neves et al., 2012) and it is rapidly downregulated as hair cells differentiate. This downregulation is required for further maturation because sustained expression of Sox2 in Atoh1 expressing cells blocks the induction of hair cell markers such as Myosin-VIIa (Dabdoub et al., 2008). Six1 and its transcriptional co-activator Eya1 are expressed in the prosensory domain of the cochlea, and they bind directly to the 3′Atoh1-enh (Ahmed et al., 2012a). These two factors are sufficient to induce Atoh1 expression in competent regions and their activation is potentiated by Sox2 (Ahmed et al., 2012a). As described before, Neurog1 is also upregulated by Six/Eya with Sox2 (Ahmed et al., 2012b).

In summary, the crucial elements in setting neuronal and sensory competence are: (1) FGF signaling that induces the expression of Sox2 in the neurosensory domain of the otic placode, (2) Sox2 that drives the activation of neuronal and sensory master genes Neurog1 and Atoh1 and neurosensory competence, and (3) the onset of Neurog1 and Atoh1 and the determination of neurons and hair cells, respectively.

## Neurons vs. hair cells: how does Neurog1 counteract Atoh1?

Although Sox2 is able to activate both Neurog1 and Atoh1, Atoh1 expression remains undetected until HC differentiation (Figure 2C). Neves et al. (2012) hypothesized that this delay in Atoh1 expression may be explained by an incoherent feed-forward loop (I-FFL) triggered by Sox2, where Sox2 activates both Atoh1 and Atoh1 repressors (Neves et al., 2012). Recent experiments using a conditional gain of function system in mouse support the model by showing that Sox2 is required for prosensory specification, but it must be downregulated to allow Atoh1 expression (Puligilla and Kelley, 2017). Major candidates to mediate the repression of Atoh1 include a variety of bHLH factors that are expressed throughout ear development such Neurog1 or Notch targets.

There is a mutual antagonism between Neurog1 and Atoh1 functions. Neurog1 null mice show a loss of sensory neurons, smaller sensory patches, and premature development of hair cells (Matei et al., 2005). Moreover reduced Neurog1 causes ectopic Atoh1 expression and that excess of Atoh1 suppresses Neurog1 (Raft et al., 2007). During development, Neurog1 overrides Atoh1 expression. Therefore, the functional antagonism between Neurog1 and Atoh1 is resolved in favor of Neurog1, the result being that neurons develop prior to hair cells. The molecular mechanism of this dominance of Neurog1 over Atoh1 is still unknown, but it seems crucial for understanding the timing of cell fate during ear development. Neurog1 is a transcriptional activator, suggesting that the counteractive interaction with Atoh1 is likely complex. In principle, Neurog1 may repress Atoh1 by the following mechanisms: (1) by preventing Atoh1 transcription, (2) by preventing Atoh1 mRNA translation, or (3) by post-translational mechanisms that result in modified Atoh1 protein levels or activity.

### Transcriptional repression of Atoh1

Neurog1 and Atoh1 are two bHLH type II proteins (Massari and Murre, 2000). They are known to dimerize with type I bHLH like E47 and bind to E-box sequences resulting in activation of transcription (Jarman et al., 1993; Koyano-Nakagawa et al., 1998; Bertrand et al., 2002). One simple possibility for Neurog1 acting as a repressor of Atoh1 is that Neurog1 acts as a partial agonist for Atoh1. Neurog1 would compete for the class A E-box located in the 3′Atoh1-enh, resulting in a weak activation but impeding the stronger autoactivation by Atoh1. Atoh1 and Neurog1 may also compete for the same E-protein partners, like E47, the result being that Atoh1 is unable to bind DNA.

Neurog1 may repress Atoh1 transcription in an indirect manner, by activating transcriptional repressors of Atoh1. Among the targets of Neurog1, NeuroD is one major effector of Neurog1 in the ear, being essential for neuroblasts delamination (Ma et al., 1996; Huang et al., 2000; Kim et al., 2001) and for shutting down Sox2 expression in the neurons (Evsen et al., 2013). Conditional NeuroD deficient mice show that NeuroD suppresses Atoh1 expression in auditory-vestibular neurons as indicated by the ectopic expression of Atoh1 after NeuroD deletion (Jahan et al., 2010). However, during early stages of neurosensory development, Neurog1 is expressed homogeneously in the neurosensory epithelium, including hair cell precursors (Raft et al., 2007), and only those cells that express high levels of Neurog1 trigger lateral inhibition and delaminate from the epithelium. Therefore, it is likely that alternative mechanisms may prevent Atoh1 without necessarily driving neuronal differentiation (Sun et al., 2001; Fritzsch et al., 2006).

### Post-transcriptional regulation of Atoh1: mRNA processing and stability

The half-life of many mRNAs can fluctuate during development and mRNA stability depends on RNA-binding proteins that bind mRNAs (Day and Tuite, 1998; Knuckles et al., 2012). Also micro-RNAs (miRNAs) are known regulators of mRNA stability or translation efficiency and modify protein expression levels. Some miRNAs like the miR-183 family (miR-96, miR-182, and miR-183) are expressed at high levels in young hair cells and ganglion neurons (Weston et al., 2006; Li et al., 2010) and the manipulation of miR-183 levels modify the number of hair cells (Li et al., 2010, see Groves et al., 2013 for a review).

Regulation of translation and protein synthesis depends on initiation factors (eIFs), some of which are phosphoproteins susceptible of regulation (Day and Tuite, 1998), but little is known about their behavior during embryonic development. Those are potential candidates to regulate the reduction of Atoh1 induced by Neurog1; however, we have no information on whether they are modified by Neurog1.

### Post-translational regulation: Atoh1 activity and degradation

Degradation of bHLH proteins has been extensively documented in different model systems and it is accounted by phosphorylations in their C-terminus domain (Forget et al., 2014; Hardwick and Philpott, 2015; Quan et al., 2016). Atoh1 post-transcriptional downregulation has been reported during cerebellar granule neuron differentiation, where BMP2 and BMP4 inhibit proliferation and induce differentiation through proteosome mediated degradation of Atoh1 (Zhao et al., 2008). BMPs induce the expression of Id1 and Id2 that upon dimerization with Atoh1 target the complex for degradation. In cerebellar granule neuron progenitors, Shh prevents Atoh1 degradation by preventing the recruitment of Atoh1 by Huwe3, an E3 ligase (Forget et al., 2014). Atoh1 is degraded by the proteosome pathway when dimerizing with Huwe1 in HEK cells, and the conditional deletion of Huwe1 generates supernumerary HCs in the mouse cochlea (Cheng et al., 2016).

Atoh1 contains in the C-terminus a potential PEST sequence (Jarman et al., 1993). This is a peptide sequence rich in proline (P), glutamic acid (E), serine (S), and threonine (T). This sequence is associated with proteins that have a short intracellular half-life and it is hypothesized that the PEST sequence acts as signal for protein degradation. Atoh1 protein stability is very short and it is extinguished in 2 h after protein synthesis blockade (Forget et al., 2014; Cheng et al., 2016). Aminoacid residues located at the C-terminus region of Atoh1 protein that are susceptible to phosphorylation are conserved among different species (Mulvaney and Dabdoub, 2012). Like Atoh1, other bHLH proteins as Neurog2 and NeuroD4 are also less stable upon phosphorylation (Hindley et al., 2012; Hardwick and Philpott, 2015).

**Figure 3:**
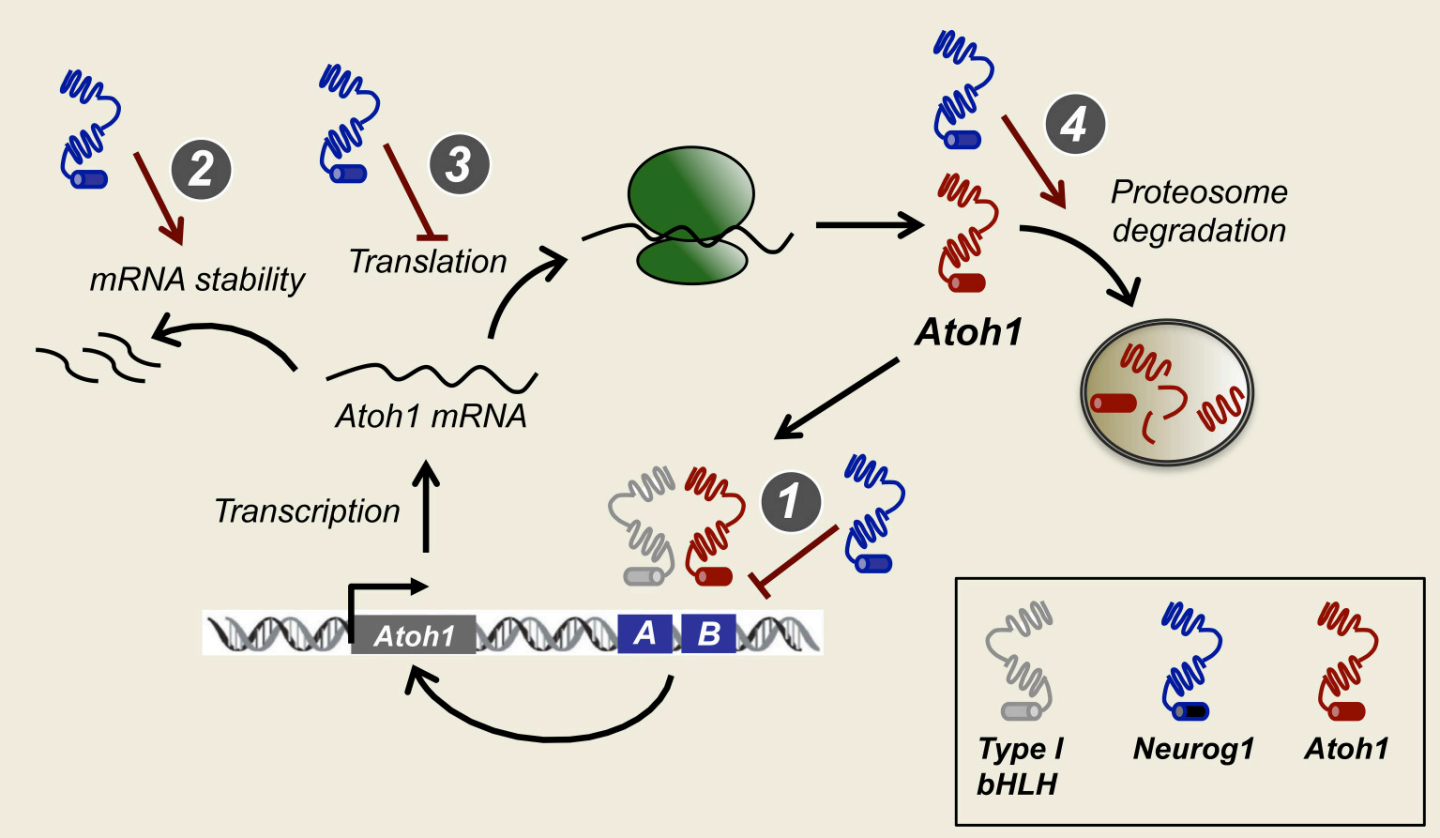
**How does Neurog1 force neurogenesis before sensorigenesis?** Possible models for Neurog1 repression of Atoh1. Neurog1 prevents Atoh1 activation by binding to the CAN region of the 3′Atoh1 enhancer or by sequestering type I bHLH factors necessary for Atoh1 binding (1). Neurog1 reduces Atoh1 protein levels by mRNA degradation (2), inhibition of protein synthesis (3), or reducing protein stability by promoting Atoh1 protein to proteosome degradation (4), of other bHLH in the prosensory epithelia.

Cyclin-dependent kinases (Cdks) drive cell cycle progression and are known to target Serine Proline (SP) and Threonine Proline (TP) sites (Errico et al., 2010). bHLH proteins like Atoh1 and Neurog1 contain several putative ST and TP in their C and N-terminal regions. Phosphorylation in these SP/TP residues may be crucial for regulating activity and is linked to the cell cycle. For example, in Xenopus embryos and P19 cells, progenitor cells that divide rapidly show Neurog2 phosphorylation and degradation, whereas when cell cycle is lengthened, Neurog2 accumulates and activates down-stream targets (Ali et al., 2011). Cell cycle exit in the cochlea is dependent on the expression of the cyclin inhibitor p27kip (White et al., 2006), and it is possible that Atoh1 is degraded in dividing prosensory progenitors until p27kip expression and cell cycle withdrawal. One plausible mechanism is that Atoh1 protein is degraded in the presence of Neurog1. This type of regulation has been recently described for Atoh1 protein when targeted by the E-3 ubiquitin ligase Huwe1 (Cheng et al., 2016). Finally, Neurog1 may also interfere with Atoh1 translation (see Figure 3).

## Notch signaling and the singling out of hair cells

Notch signaling is an evolutionarily conserved juxtacrine signaling pathway used by metazoans. It controls a broad spectrum of developmental processes in organisms ranging from sea urchins to humans (Artavanis-Tsakonas et al., 1999; Neves et al., 2013). Lateral inhibition is one major operation mode of the pathway by which a ligand-producing cell signals its neighbors to reduce ligand expression (see (Neves et al., 2013) for review on the different modes of operation of Notch during ear development). Lateral inhibition mediates binary cell fate decisions by ensuring that the cells adopt one of two alternative fates. In the inner ear Notch mediates the determination of two major cell types, neurons and hair cells. Driven by Sox2, progenitors residing in the neurosensory domain express Neurog1, some of them with enough strength so to unfold the neuronal program and become neuroblasts. Nascent neuroblasts express the ligand Delta-like1 (Dll1), which activates Notch1 in the neighboring cells and suppress Neurog1 expression. Neuroblasts delaminate, and the cells that remain in the neurosensory epithelium are fated to become sensory precursors (the prosensory patches). Later in development, some cells from the prosensory patches start expressing Atoh1, which initiates a second round of lateral inhibition by which some precursors activate the ligands Delta1 (in mammals also Jag2) that drive lateral inhibition. The result is that those cells that express Atoh1 become hair cells and prevent the neighbors to do so, generating the typical mosaic of alternate cell types (Adam et al., 1998; Eddison et al., 2000; Neves et al., 2013).

Both during neurogenesis and hair cell generation, the action of Notch ligands results in the expression of the typical Notch targets like Hes and Hey factors (Petrovic et al., 2014, 2015). The most studied Notch canonical effectors are Hairy and Enhancer of Split (Hes) and Hairy and enhancer of split related (Hey). Hes and Hey genes belong to the type VI bHLH group. Seven Hes members have been identified in vertebrates (Hes1–7), while the Hey subfamily of genes encodes three members in mammals (Hey1, Hey2, and HeyL; Iso et al., 2001, 2003).

The core structure of Hes and Hey proteins contains a basic and Helix-loop-Helix domain and an Orange domain at the C-terminus region. The Orange domain serves as a region for protein-protein interactions and for partner selection (Iso et al., 2001). Hey proteins differ from the Hes subgroup by two striking features: first a glycine present in the basic domain of Hey proteins instead of a conserved proline in Hes proteins, which confers DNA-binding specificity (Leimeister et al., 1999). Secondly, the C-terminal WRPW motif that is characteristic of Hes proteins and allow Groucho co-repressor recruitment, is replaced with YRPW or YXXW (HeyL; Fisher et al., 1996). The C-terminal WRPW of Hes motif acts as polyubiquitination signal, making Hes proteins short-living (Hirata et al., 2002; Iso et al., 2003).

Hes factors bind with high affinity to E-box class C or N-box. Hey1, due to the presence of a glycine residue in the basic domain has preference to class C or class B E-boxes (Iso et al., 2003). The repressive function can be either active or passive. Active repression involves DNA binding, whilst in passive repression Hey/Hes proteins sequester bHLH type I family and impair their heterodimerization with class II bHLH (Iso et al., 2003).

During development, several Hes and Hey genes are expressed in the inner ear. Hes5 is the major Notch target expressed during lateral inhibition. It is detected in the precursors that are not selected as neurons or hair cells. Its expression correlates well with that of Dll1 in nascent neurons and hair cells (Petrovic et al., 2014). Hey1 is also expressed in the prosensory epithelium, concomitantly with Jagged1, and co-expressed with Hes5 during hair cell formation (Petrovic et al., 2014). Although Hey1 and Hes5 are direct Notch downstream targets, they differ in the level of Notch required for their activation.

Knockout mice of different Hey and Hes factors exhibit supernumerary hair cells in the cochlea, suggesting a repressor function during hair cell development. The combined loss of function of Hes5, Hey1, and Hes1 results in supernumerary hair cells (Tateya et al., 2011) and Atoh1 is upregulated after interference of Hey1/Hes5 expression with siRNAs (Du et al., 2013) or treatment with the Notch inhibitor DAPT (Ren et al., 2016). Notch inhibition of damaged sensory epithelia favors HC regeneration (Lin et al., 2011; Mizutari et al., 2013), suggesting that these factors may also regulate the ability to regenerate HCs (see below).

## The repression of Atoh1 by hes and hey: is it all in the 3′Atoh1 enhancer?

**Figure 4:**
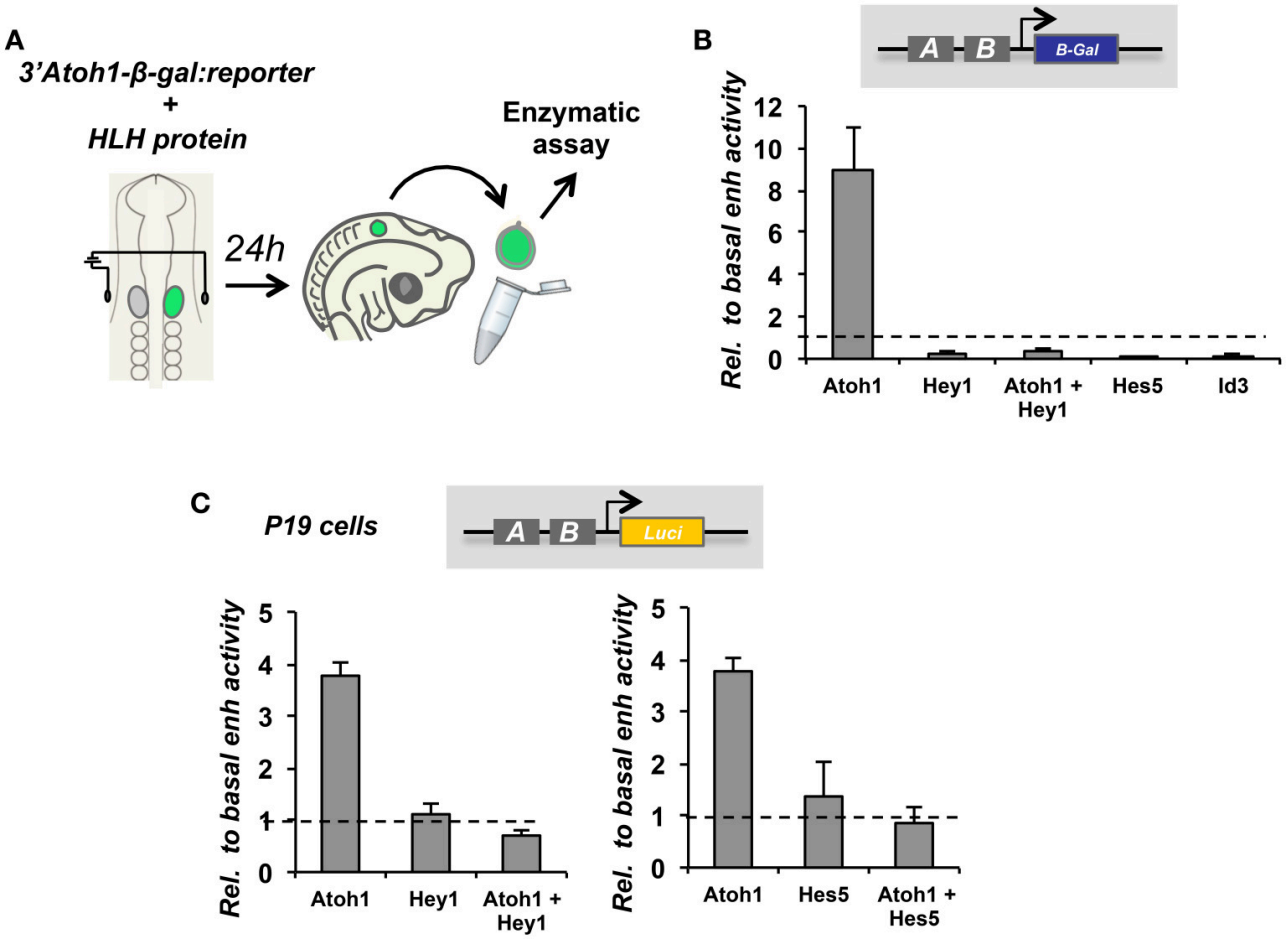
**3′Atoh1 enhancer regulation by the Notch targets, Hey1 and Hes5. (A)** Schematic representation of in ovo chicken electroporation of the 3′Atoh1 enhancer reporter in combination with bHLH factors. **(B)** Otic vesicles were isolated and developed in ovo for 24 h after in ovo electroporation (E2+1). Reporter β-gal activity measured in the conditions indicated (*n* = 3–4). Levels of electroporation were normalized by luciferase activity. Atoh1 activated its own enhancer and Hey1 prevented Atoh1 autoactivation. All three bHLH factors repressed 3′Atoh1 enhancer basal activity. **(C)** Hey1 (left) and Hes5 (right) also prevented Atoh1 autoactivation in P19 cells. Values of luciferase correspond to enhancer activity relative to the basal activity of the 3′Atoh1 enhancer in the conditions indicated in abscissa (*n* = 3). Values are normalized by renilla activity. Data displayed as Mean + S.E.M.

**Figure 5:**
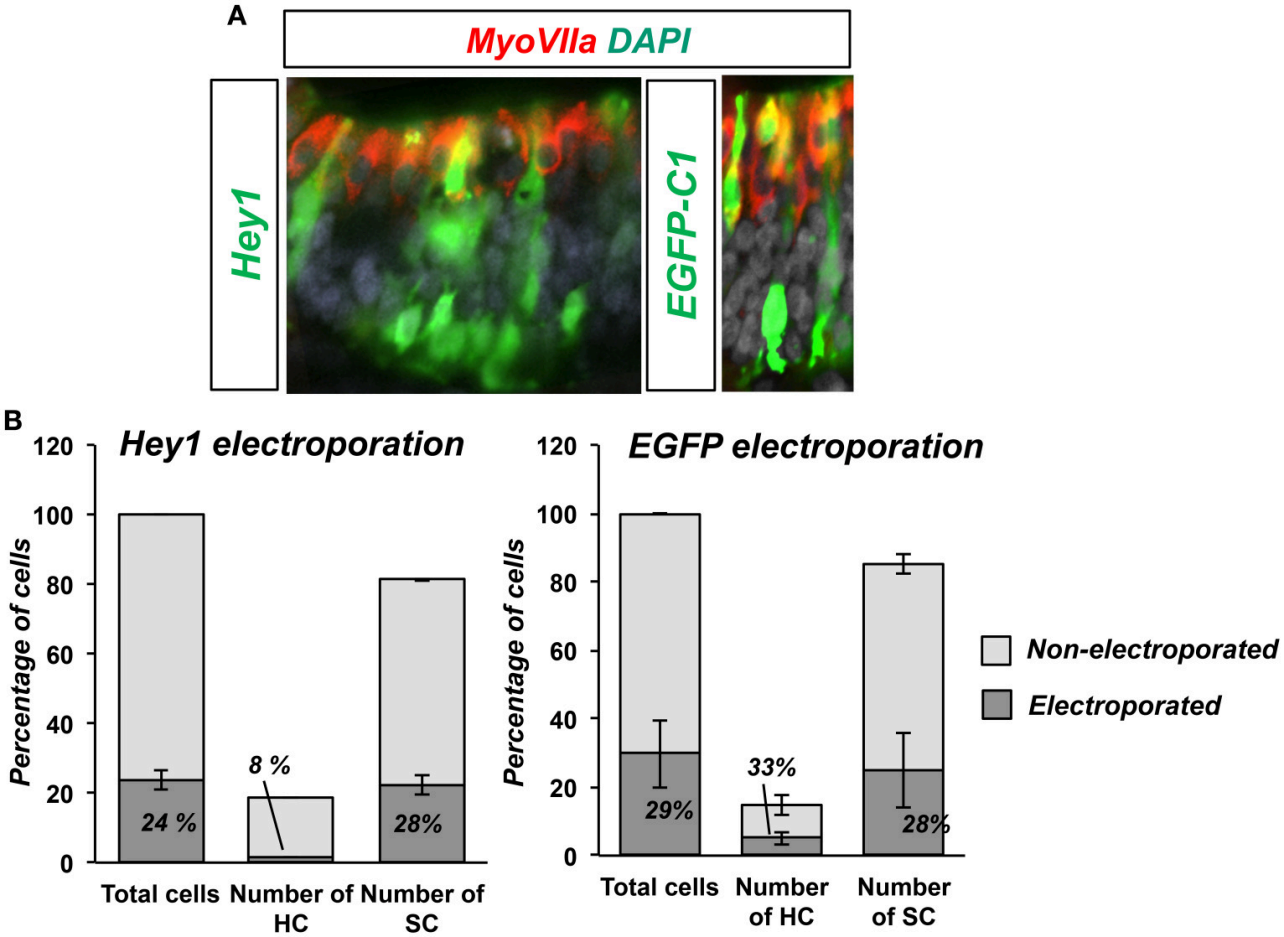
**Hey1 prevents HC formation in ovo. (A)** E3.5 chicken embryos were electroporated with Hey1 (left image) or EGFP-C1 (right image) and then sectioned after 3 days of incubation (E3.5+3). Electroporated cells in the macula sacularis were found mainly in the SC layer, and very few developed as HCs. Control electroporation with EGFP-C1 (E3.5+3) is shown on the right. **(B)** Hey1 electroporation biased electroporated cells toward supporting cell fate. The fraction of HCs that were electroporated (8%) was smaller than that of SCs (28%), similar to the efficiency of the electroporation (24%). Bars represent the number of cells counted in two consecutive frames of electroporated macula sacularis, from three independent embryos (*n* = 3). Electroporation with EGFP-C1 did not show any bias for either HCs or SC.

Hey1 and Hes5 repress Atoh1 and silence the 3′Atoh1-enh (Figures 4A,B). In addition, both factors are able to block Atoh1 autoactivation (Figure 4C), suggesting that the repression of Atoh1 by Hes5 and Hey1 prevails upon its own activation. Accordingly, and parallel to 3′Atoh1-enh repression, Hey1 overexpression is sufficient to prevent HC generation in chick sensory epithelia (Figure 5). Taken together, these observations suggest that during development, Notch targets Hey1 and Hes5 act on the 3′Atoh1-enh and repress Atoh1 expression in prosensory precursors and supporting cells.

**Figure 6:**
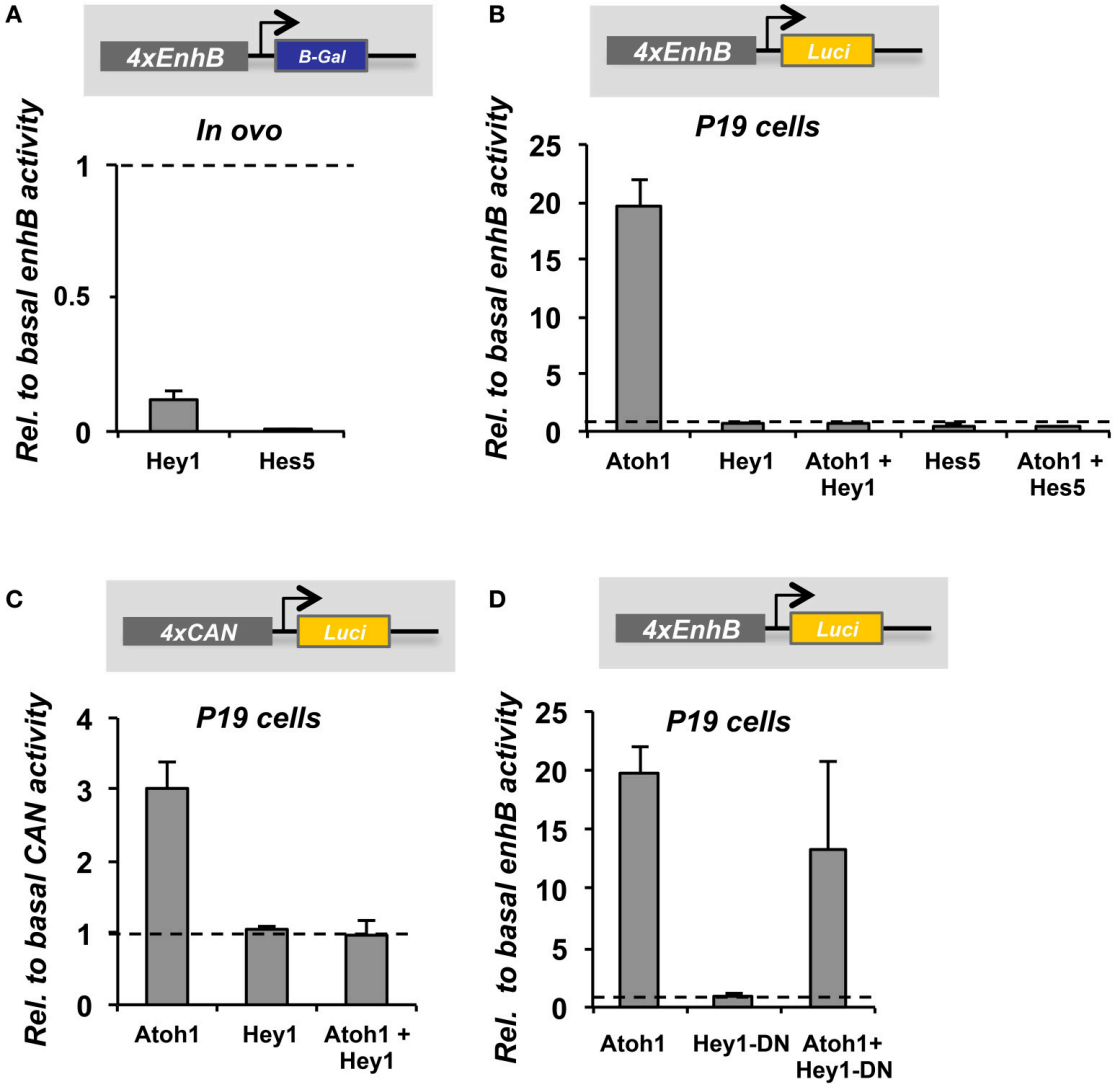
**The regulation of the 3′Atoh1 enhancer by Hey1 and Hes5 is recapitulated by the CAN region**. **(A)** Quantification of EnhB activity in the presence of Hey1 or Hes5 in E2+1 otic vesicles. Hey1 and Hes5 factors were able to prevent the basal activity of 4 × EnhB (*n* = 3). **(B)** In P19, Atoh1 was able to activate 4 × EnhB and the autoactivation was suppressed by either Hey1 or Hes5 (*n* = 3). **(C)** The CAN multimer was activated by Atoh1, but Hey1 was not able to repress the basal reporter activity. However, it prevented Atoh1 autoactivation (*n* = 3). **(D)** Hey1 requires its DNA binding domain to repress the CAN region. Quantification of 4 × EnhB activity with Atoh1 and Hey1-DN (Hey1 dominant negative) in P19 cells. Data displayed as Mean + S.E.M.

Hey1 is also able to prevent both, the basal activity and the autoactivation of EnhB of the 3′Atoh1-enh in chicken otic vesicles and P19 cells (Figures 6A,B). Hes5 can also prevent Atoh1 autoactivation (Figure 6B). Moreover, the importance of the CAN region of EnhB is illustrated by multimer reporter analysis showing that Hey1 requires the E-boxes flanking the class A E-box to act as a repressor (Figure 6C). The CAN multimer mimics the 3′Atoh1-enh repression promoted by Hey1, indicating that the minimal region of the enhancer to explain the repression is the CAN region.

**Figure 7:**
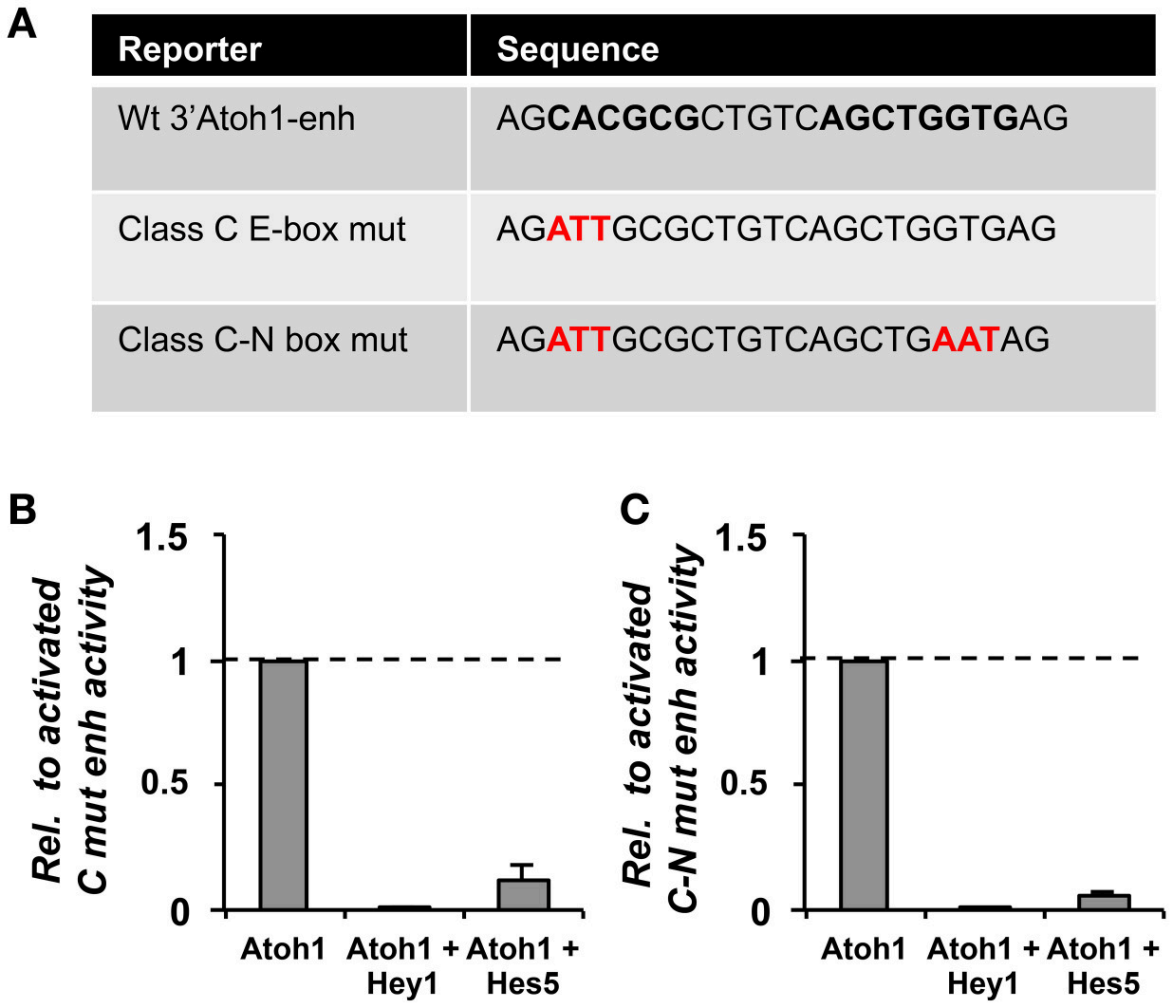
**Hey1 and Hes5 predicted binding sites were not enough to prevent the repression of the 3′Atoh1 enhancer. (A)** Table showing the mutations generated in the E-boxes of the CAN region (or located in enhancer B). The three E-boxes are depicted in black and bold, in red the mutated nucleotides. **(B)** Hey1 and Hes5 were able to block Atoh1 autoactivation even after mutation of the class C E-box (*n* = 3). **(C)** Similarly, the simultaneous mutation of the E-box C and N-box had no effect on the repression of the 3′Atoh1 enhancer (*n* = 2, Hey1, and *n* = 3, Hes5). Data displayed as Mean + S.E.M.

Hey1 needs to bind DNA in order to repress Atoh1, since the mutation of Hey1 DNA binding domain abolishes repression (Figure 6D). However, the identification of the region bound by Hey1 and Hes5 has been difficult and still remains elusive. On the one hand, mutations of either the class C E-box or the N-box of the CAN region are unable to prevent Atoh1 repression by Hey1 and Hes5 (Figure 7). This is in agreement with the results of ChIP-seq analysis performed on HEK 293, which shows that Hey1 does not bind to the 3′Atoh1-enh (Heisig et al., 2012). But it is also somehow surprising and suggests that there are alternative binding sites and/or mechanisms of repression for Hes and Hey factors. For example, it is possible that Hey1 blocks the transcription of Atoh1 by interfering with the class A E-box. This possibility is difficult to explore since the mutation of E-box A silences the 3′Atoh1-enh.

A recent study has shown that Hes5 and Hey2 are able to prevent Atoh1 expression by binding to the promoter region of Atoh1, and that the repression of Atoh1 in supporting cells depends on this interaction, rather than on the 3′Atoh1 enhancer, which would operate mainly for Atoh1 activation (Abdolazimi et al., 2016). Gene repression and binding by Hey1 is also dependent on the chromatin signature of the promoter regions. Heisig et al. (2012) found that Hey1 bound sequences overlapped with the presence of polymerase II and the active chromatin mark H3K4m3, characteristic of active and poised promoters. H3K4m3 chromatin marks are found in Atoh1 promoter and enhancer prior to Atoh1 upregulation (Stojanova et al., 2015). Therefore, the regulation of Atoh1 is likely to be dependent on multiple sites, and not only mediated by the 3′Atoh1 enhancer. The relationship between the Enhancer and promoter regions may be crucial to fully solve the complex regulation by Hey1.

## From development to regeneration

Hearing loss is a major problem affecting more than 360 million people in the industrialized world (WHO). It affects speech and language and leads to severe deficits in communication, and a strong negative impact in the quality of life. Hearing impairment is mainly caused by the failure of hair cells and/or otic neurons (sensorineural hearing loss), hair cell damage being the most frequent triggering factor. Hair cell damage arises from genetic defects, aging, noise, traumatic lesions, infections, or therapeutic substances. The main problem of hair cell damage is that, unlike other animal species, mammals are not able to regenerate hair cells of the auditory epithelia and there is no treatment for hearing deficiencies in humans.

In contrast to mammals, non-mammalian vertebrates like chicken, zebrafish, or lizards, are able to repair and heal damaged sensory epithelia. In the chick, damaged hair cells trigger supporting cells to replace lost hair cells by two different mechanisms: (1) mitotic regeneration, where SC divides asymmetrically and one daughter cell remains as SC and another as HC, and (2) transdifferentiation of SC into HC. In transdifferentiation, HCs are generated at the expense of SCs, which become exhausted and hence, the epithelium is disorganized. The consequence is that, although HCs are recovered, hearing function is not (Stone and Cotanche, 2007). In birds, hair cell regeneration starts with direct transdifferentiation of SCs into HCs, followed by mitotic regeneration and the correct replacement of the sensory epithelium and auditory function (Roberson et al., 2004).

Although mammals have some capacity to regenerate hair cells in the vestibular organs and the early post-natal cochlea, the adult auditory organ is completely devoid of this capacity. The question arises as to what are the differences between birds and mammals that explain their different regenerative capacity. Are there signals that regulate SC quiescence and activation after HC loss in chicken? Are they similar to mammalian early post-natal regeneration? Why mammals lose the capacity of regeneration after birth?

Studies in the chick have shown that hair cell regeneration reuses mechanisms that operate during embryonic development. Several molecular pathways known to regulate embryonic hair cell progenitors are reactivated in mature chicken epithelia after HC loss. Upon HC damage, Atoh1 becomes reactivated in transdifferentiating and mitotically active SCs (Cafaro et al., 2007). Atoh1 reactivation is essential to form new hair cells, like it is to form hair cells during development (Bermingham, 1999). Notch signaling is down-regulated upon damage in the basilar papilla suggesting that in the mature organ it maintains a repressive state that prevents Atoh1 expression. In agreement, different laboratories have shown that treatment with Notch inhibitors favors Atoh1 reactivation and HC regeneration in the chick basilar papilla and also in the post-natal mammalian cochlea under certain conditions (Cafaro et al., 2007; Mizutari et al., 2013). The ability of SC to respond to Notch blockade dramatically declines after birth, and is lost by post-natal day 6 (Maass et al., 2015).

Human stem cells constitute a reasonable alternative to replace damaged hair cells. Major problems of this approach are the difficulty to deliver treated cells to the damaged areas and their limited ability to integrate in the epithelium. Several groups have developed protocols to differentiate hair cells by mimicking the hair cell development in the embryo. Although this has proved successful, the efficiency of the procedures in hair cell regeneration is still very low (Chen et al., 2012; Ronaghi et al., 2014). In contrast to the low efficiency in replacing hair cells, stem cell therapy has proven surprisingly effective at restoring auditory neurons. The first reports of otic guidance with monolayer cultured human ESCs (hESCs) revealed a propensity to differentiate along an otic neurogenic lineage rather than HC lineage (Chen et al., 2012). Different attempts to generate HCs in culture commonly face the problem that most cells go to the neuronal cell fate, making it very difficult to enrich them in HCs (Chen et al., 2012; Ronaghi et al., 2014). This problem is directly related to the question addressed in the present work. During early stages of development, Neurog1 prevails over Atoh1, thereby forcing neurogenesis and delaying sensorigenesis. This suggests that the default fate is to become a neuron and that sensory competence is silenced. If this is so, the consequence is that production relies mainly on relieving the repression of hair cell competence rather than on the expression of activators. The cellular context of conditionally derived stem cells may be similar to that in the embryo and interference with Neurog1 may open a way to improve the efficiency of HC production.

In summary, understanding the developmental mechanisms involving interactions among cell-to-cell signals and transcription factors is crucial for designing strategies for hearing repair. Developmental studies have shown that the connection between FGF signaling and neurosensory commitment relies on the induction of SoxB1 factors, which set the expression of Neurog1 and Atoh1. The further interaction between Neurog1 and Atoh1 is crucial for neuronal and hair cell specification and for setting the timing for cell diversification. Notch operates at several stages during ear development, but one of them is linked to proneural gene expression and the irreversible commitment to neuronal and hair cell fate of only a fraction of the competent progenitors. Its function is crucial for maintaining dormant the potential of prosensory cells to develop as hair cells, and to keep the regenerative potential of supporting cells. Further work is required to better understand the details of the molecular mechanisms of Atoh1 and Neurog1 regulation, and the development and regeneration of neurons and hair cells.

## Ethics statement

The experiments reported in this paper were carried out on E2-E3 chick embryos according to national regulations following a protocol approved by the Ethics Committee of the PRBB.

## Author contributions

HG, GA, and FG: Planning, executing, and writing.

## Funding

La Marató and BFU2015-67400-P (MINECO/FEDER, UE).

### Conflict of interest statement

The authors declare that the research was conducted in the absence of any commercial or financial relationships that could be construed as a potential conflict of interest.
